# Mechanisms of Mitral Regurgitation: New Insights From 3D Echocardiography and Implications for Transcatheter Repair

**DOI:** 10.1016/j.jacasi.2021.04.004

**Published:** 2021-05-21

**Authors:** Alex Pui-Wai Lee, Yiting Fan, Faisal N. Baig, Yat-Yin Lam

Mitral regurgitation (MR) is classified according to its etiology into primary/degenerative (DMR) or secondary/functional (FMR). Several transcatheter mitral valve (MV) repair (TMVr) techniques are available or under investigation. The MitraClip, a transcatheter edge-to-edge leaflet repair (TEER) system, is currently the only approved TMVr device in Asia. In this paper, we discuss with examples, novel MR mechanisms revealed by 3-dimensional (3D) echocardiography and their implications for TMVr.

**Figure 1:**
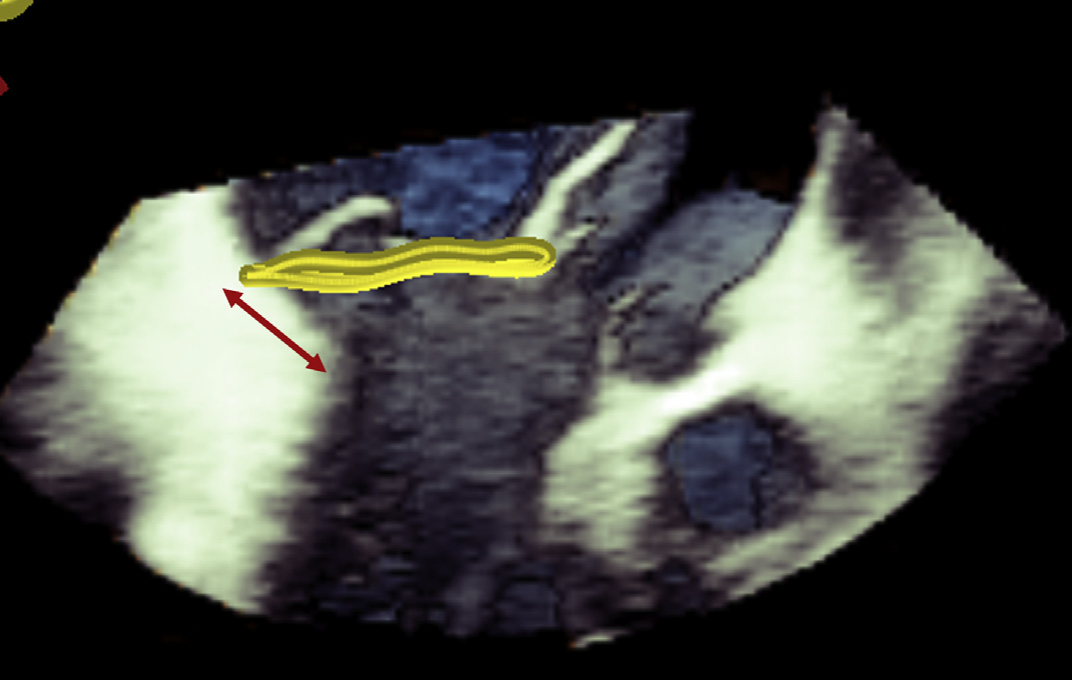
Mitral Annular Disjunction and Unsaddling in Primary/Degenerative Mitral Regurgitation Transcatheter edge-to-edge leaflet repair may not restore decoupled annular function in mitral annular disjunction **(arrow)**.

The normal mitral annulus motions consist of: 1) a sphincter-like contraction that reduces area especially in the anteroposterior (A-P) direction; and 2) saddle-shape deepening that reduces systolic leaflet/chordal stress (1). Motions of the fibrous annulus are translationally driven by contraction of left ventricle (LV) and left atrium (LA) fibers (annulo-atrio-ventricular coupling). Mitral annular disjunction (MAD) is a separation of the LA-MV junction from the LV attachment (Figure 1) that is frequently observed in DMR (2). MAD functionally decouples the mitral annulus from LV contraction. Paradoxical systolic annular expansion worsens MR by reducing leaflet coaptation; moreover, systolic annular unsaddling accentuates leaflet/chordal stress, perpetuating leaflet prolapse, chordal rupture, and fibrosis predisposing to arrhythmias. TEER reduces MR by improving leaflet coaptation and creating a tissue bridge that limits annular A-P dilation. We speculate that TEER may not be able to effectively restore decoupled annular function, because annulus-leaflet-ventricular tissue continuity is lost in MAD. Progressive degeneration and proarrhythmic substrates may persist. In this case, an annuloplasty-based strategy may be preferred. This is an important consideration for expanding indications of TMVr in more complex lesions and younger patients. In addition, 3D echocardiography is important for treating commissural lesions in which nonclassical x-plane is used for guiding leaflet grasping (3).

In ventricular FMR, the mitral annulus is dilated and fractional changes of circumference and area are reduced (2). In response to annular dilation and/or leaflet tethering (4), leaflet area grows larger as a compensatory mechanism. Adaptive leaflet growth, however, fails to compensate when the leaflet-to-closure area ratio falls below 1.4–1.7 (4). Ventricular tethering of mitral leaflets can be symmetrical (apical) or asymmetrical (posterior) (5). Regional inferior remodeling displaces papillary muscles posteriorly, resulting in asymmetrical tethering; global remodeling tethers both leaflets apically. Success in MR reduction with annuloplasty is good in basal but low in distal anterior leaflet tethering (distal anterior leaflet angle >25°) (5). In this case, transcatheter annuloplasty, analogous to its surgical counterpart, may be more suitable for patients with no or asymmetrical posterior tethering, but not for those with symmetrical apical tethering. The tissue bridge created by TEER can produce symmetrical tension on both leaflets that shortens A-P diameter and counterbalances ventricular tethering forces. Symmetrical tethering allows better clip trajectory for leaflet grasping than asymmetrical tethering, which may require independent grasping. Symmetrical leaflet remodeling with uniform growth of leaflet lengths may also favor leaflet insertion. However, insufficient leaflet remodeling as assessed by total leaflet/annular area ratio is associated with residual MR ≥2+ after TEER, suggesting that 3D MV geometry should be considered in patient selection (6). In addition, identification of leaflet clefts/deep indentations on 3D echocardiography may prompt the use of advanced clip implantation techniques (3).

FMR occurring in patients with atrial fibrillation/myopathy with normal LV ejection fraction has been termed atrial FMR. Normally, atriogenic presystolic annular contraction accounts for 60% of total contraction and brings the leaflets together before systolic LV pressure rise; systolic ventriculogenic annular contraction continues to sustain leaflet coaptation throughout systole (7). In atrial fibrillation/myopathy, atriogenic annular contraction is diminished but rarely causes significant MR. Recent 3D and strain echocardiographic studies found that atrial FMR is associated with impaired LV longitudinal strain (7) that diminishes systolic annular contraction, contributing to MR. Atriogenic leaflet tethering, leaflet growth imbalance, and LA hypertension also contribute to atrial FMR (4,7). Unlike ventricular FMR, clinical benefits of TEER for atrial FMR have yet to be established. TEER may be more effective in reducing annular A-P diameter in atrial FMR with normal leaflet motions than in ventricular FMR with restricted leaflet motion, because leaflet captures are parallel to the A-P instead of the sagittal direction (8). However, atriogenic leaflet tethering may shorten leaflet length for leaflet capture.

The mechanisms of MR are complex and heterogeneous. Understanding novel MR mechanisms and their implications for TMVr is important for clinical practice.
